# Functional motor phenotypes: to lump or to split?

**DOI:** 10.1007/s00415-021-10583-w

**Published:** 2021-05-07

**Authors:** Michele Tinazzi, Christian Geroin, Enrico Marcuzzo, Sofia Cuoco, Roberto Ceravolo, Sonia Mazzucchi, Andrea Pilotto, Alessandro Padovani, Luigi Michele Romito, Roberto Eleopra, Mario Zappia, Alessandra Nicoletti, Carlo Dallocchio, Carla Arbasino, Francesco Bono, Giuseppe Magro, Benedetta Demartini, Orsola Gambini, Nicola Modugno, Enrica Olivola, Laura Bonanni, Elisabetta Zanolin, Alberto Albanese, Gina Ferrazzano, Rosa De Micco, Leonardo Lopiano, Giovanna Calandra-Buonaura, Martina Petracca, Marcello Esposito, Antonio Pisani, Paolo Manganotti, Lucia Tesolin, Francesco Teatini, Tommaso Ercoli, Francesca Morgante, Roberto Erro

**Affiliations:** 1 Neurology Unit, Movement Disorders Division, Department of Neurosciences, Biomedicine and Movement Sciences, University of Verona, P.le Scuro 10, 37134 Verona, Italy; 2 Center for Neurodegenerative Diseases (CEMAND), Department of Medicine, Surgery and Dentistry-Scuola Medica Salernitana, University of Salerno, Baronissi, SA Italy; 3 Neurology Unit, Department of Clinical and Experimental Medicine, University of Pisa, Pisa, Italy; 4 Neurology Unit, Department of Clinical and Experimental Sciences, University of Brescia, Brescia, Italy; 5 FERB Onlus, Ospedale S. Isidoro, Trescore Balneario, Bergamo, Italy; 6 Parkinson and Movement Disorders Unit, Fondazione IRCCS Istituto Neurologico Carlo Besta, Milan, Italy; 7 Department G.F. Ingrassia, Section of Neurosciences, University of Catania, Catania, Italy; 8 Department of Medical Area, Neurology Unit, ASST Pavia, Pavia, Italy; 9 Botulinum Toxin Center, Neurology Unit A.O.U. Mater Domini, Catanzaro, Italy; 10 Aldo Ravelli Research Center for Neurotechnology and Experimental Brain Therapeutics, Department of Health Sciences, University of Milan, Milan, Italy; 11 IRCCS Neuromed, Pozzilli, Italy; 12 Department of Neuroscience, Imaging and Clinical Sciences, University G. D’Annunzio, Chieti-Pescara, Italy; 13 Unit of Epidemiology and Medical Statistics, Department of Diagnostics and Public Health, University of Verona, Verona, Italy; 14 Department of Neurology, IRCCS Humanitas Research Hospital, Rozzano Milan, Italy; 15 Department of Human Neurosciences, Università La Sapienza, Rome, Italy; 16 Department of Advanced Medical and Surgery Sciences, University of Campania-Luigi Vanvitelli, Naples, Italy; 17 Department of Neuroscience-Rita Levi Montalcini, University of Turin, Turin, Italy; 18 Department of Biomedical and Neuromotor Sciences, University of Bologna, Bologna, Italy; 19 IRCCS, Institute of Neurological Sciences of Bologna, Bologna, Italy; 20 Movement Disorder Unit, Fondazione Policlinico Universitario A. Gemelli IRCCS, Rome, Italy; 21 Clinical Neurophysiology Unit, Cardarelli Hospital, Naples, Italy; 22 IRCCS Mondino Foundation, Pavia, Italy; 23 Department of Brain and Behavioral Sciences, University of Pavia, Pavia, Italy; 24 Clinical Neurology Unit, Department of Medical, Surgical and Health Services, University of Trieste, Trieste, Italy; 25 Functional Movement Disorders Outpt. Clinic, Clinical Neurology and Stroke Unit Department, Central Country Hospital, Bolzano, Italy; 26 Department of Medical Sciences and Public Health, University of Cagliari, Cagliari, Italy; 27 Neurosciences Research Centre, Molecular and Clinical Sciences Neurosciences Research Centre, Molecular and Clinical Sciences Research Institute, St George’s University of London, London, UK; 28 Department of Experimental and Clinical Medicine, University of Messina, Messina, Italy

**Keywords:** Functional neurological disorders, Functional dystonia, Functional tremor, Functional weakness, Non-motor features, Psychogenic movement disorders

## Abstract

**Introduction:**

Functional motor disorders (FMDs) are usually categorized according to the predominant phenomenology; however, it is unclear whether this phenotypic classification mirrors the underlying pathophysiologic mechanisms.

**Objective:**

To compare the characteristics of patients with different FMDs phenotypes and without co-morbid neurological disorders, aiming to answer the question of whether they represent different expressions of the same disorder or reflect distinct entities.

**Methods:**

Consecutive outpatients with a clinically definite diagnosis of FMDs were included in the Italian registry of functional motor disorders (IRFMD), a multicenter data collection platform gathering several clinical and demographic variables. To the aim of the current work, data of patients with isolated FMDs were extracted.

**Results:**

A total of 176 patients were included: 58 with weakness, 40 with tremor, 38 with dystonia, 23 with jerks/facial FMDs, and 17 with gait disorders. Patients with tremor and gait disorders were older than the others. Patients with functional weakness had more commonly an acute onset (87.9%) than patients with tremor and gait disorders, a shorter time lag from symptoms onset and FMDs diagnosis (2.9 ± 3.5 years) than patients with dystonia, and had more frequently associated functional sensory symptoms (51.7%) than patients with tremor, dystonia and gait disorders. Patients with dystonia complained more often of associated pain (47.4%) than patients with tremor. No other differences were noted between groups in terms of other variables including associated functional neurological symptoms, psychiatric comorbidities, and predisposing or precipitating factors.

**Conclusions:**

Our data support the evidence of a large overlap between FMD phenotypes.

**Supplementary Information:**

The online version contains supplementary material available at 10.1007/s00415-021-10583-w.

## Introduction

Functional motor disorders (FMDs) consist of involuntary movements, posturing, gait disorder and weakness, which are inconsistent and incongruent with recognized neurological diseases [1]. In clinical practice, FMDs are usually categorized according to the predominant phenomenology, as this approach facilitates the diagnostic work-up and choice of symptomatic therapeutic options. However, it is unclear whether this phenotypic classification mirrors the underlying pathophysiologic mechanisms of the disorders. Whereas prior studies have provided contradictory results regarding possible clinical differences between psychogenic non-epileptic seizures (PNES) and FMDs (for a review see [2]), only one study has attempted to compare different motor phenotypes within the group of FMDs [3]. Indeed, Gelauff et al. have compared a number of features including demographics, mode of onset, mood dysfunction such as depression and anxiety, and presence of pain and fatigue between different FMDs phenotypes and failed to identify any differences, suggesting a shared pathophysiology between these disorders [3]. However, in most cases, patients had a complex syndrome consisting of two or more FMDs and the stratification was based on the ‘predominant’ symptom as judged by the referring clinicians either provided directly on request or via their clinical letter, which has likely introduced an evaluation bias. It has been shown that there is poor to moderate agreement, even between experienced movement disorder specialists, on the clinical diagnosis of functional hyperkinetic movements [4]. Their results could be also partly biased by the fact that data were collected through online questionnaire and this might have led to an under- or over-reporting of some aspects. Finally, Gelauff et al. included in their analysis patients with co-morbid neurological diseases [3], which might have further confounded the results, hampering the recognition of significant differences between FMDs phenotypes.

To overcome these possible limits, we explored the Italian registry of functional motor disorders (IRFMD) to compare clinical and demographic characteristics of patients with isolated FMDs (i.e., patients presenting with only one FMDs and without co-morbid neurological disorders, aiming to answer the question of whether they represent different expressions of the same disorder or reflect distinct entities.

## Methods

Data were extracted from the IRFMD managed by the Department of Neurosciences, Biomedicine and Movement Sciences, University of Verona, and by the Italian Academy for the Study of Parkinson’s Disease and Other Movement Disorders (Accademia LIMPE-DISMOV RADAC project) and Fondazione LIMPE. Full methods of the IRFMD are reported elsewhere [5].

Briefly, consecutive patients aged > 10 years, with a clinically definite diagnosis of FMDs according to existing clinical criteria [1] were recruited from 25 tertiary movement disorders centers across Italy.

Patients underwent a standardized evaluation to collect several information including sex, age, education, mode of onset (acute, defined as abrupt with deterioration within a few weeks [5]; or slowly progressing), previous consultations and diagnoses of FMDs (general neurologist, neurologist specialized in movement disorders), presence of predisposing and precipitating factors, presence of spontaneous remissions, time lag from onset of symptoms to FMDs diagnosis, self-reported non-motor symptoms, presence of psychiatric and medical history including certified non-neurological comorbidities, proxy of health status (calculated as the average of total number of non-neurological comorbidities), and associated functional neurological disorders (FNDs). All information were gathered by specific questions addressed to both the patients and relatives/care-givers and additionally by reviewing available medical records [5].

Presence of cognitive or physical impairment that precluded signing the informed consent form for participation in the study represented an exclusion criteria. The study was approved by the Ethics Committee of the Coordinator Centre (University of Verona, Prog. 1757CESC) and confirmed by the Committees of each participating center. All patients were informed about the nature of the study and provided their written consent to participate*.* For the aim of the current study we selected from the IRFMD patients with isolated FMDs and without co-morbid neurological disorders.

### Statistical analysis

Data are expressed as mean ± standard deviation (SD) or median and interquartile range (IQR), as appropriate, for continuous variables and counts and percentages for categorical variables. One-way ANOVA was used for normally distributed data and Kruskal–Wallis test for non-normally distributed data for group comparisons. Chi-squared test or Fisher’s exact test were used to compare categorical variables, as appropriate.

When statistical differences between groups were found with a *p*-value < 0.05, post-hoc Tukey Test was used for pairwise comparisons between groups for continuous variables. *Z*-tests of Two Proportions or Fisher’s exact tests (2 × 2) were used accordingly for the categorical ones: we applied Bonferroni correction for multiple comparison. Statistical analyses were performed using SPSS statistical software (version 26; IBM-SPSS, Armonk, NY, USA).

## Results

As of December 2020, 410 patients were included in the IRFMD [5], of whom 177 (43.2%) were identified as having an isolated FMDs and without neurological comorbidities. Of these, 1 with functional Parkinsonism was excluded and 10 with facial FMDs were included in the ‘Jerks ‘category because of their low prevalence. Therefore, 176 patients were included in the current analysis: 58 (33%) patients with weakness, 40 (22.7%) with tremor, 38 (21.6%) with dystonia, 23 (13.1%) with jerks/facial FMDs, and 17 (9.6%) with gait disorders.

The mean age of the overall cohort was 44.5 ± 16.8 years and females represented the large majority (69.3%). The overall time lag from onset of symptoms to FMDs diagnosis mean was 4.5 ± 5.1 years. Symptoms occurred in an acute onset fashion in 75% (*n* = 132) of cases.

Table 1 shows the comparisons of the main demographic and clinical variables between groups.

**Table 1:** Demographic and clinical features of different FMDs phenotypes

Variable	Total	Weakness	Tremor	Dystonia	Jerks	Gait disorders	Group comparison
Patients, *n* (%)	176	58 (33)	40 (22.7)	38 (21.6)	23 (13.1)	17 (9.6)	
Gender, *n* (%)							
Male		16 (27.6)	16 (40)	13 (34.2)	5 (21.7)	4 (23.5)	0.495^C^
Female		42 (72.4)	24 (60)	25 (65.8)	18 (78.3)	13 (76.5)	
Age, y, mean (SD)		40.1 (13.8)^**#**^ *	49.6 (18.1)	42.4 (15.8)*	40.2 (17.5)*	57.6 (15.9)	**<0.001**^A^
Education, y, mean (SD)^a^		12.1 (2.7)	11 (3.7)	12.4 (3.9)	12.3 (3.4)	9.9 (5)	0.136^K^
, median (IQR, Q1-Q3)		13 (10–13)	13 (8–13)	13 (8–15)	13 (9.5–15.8)	10.5 (5–13)	
Previous consultations, n (%)		42 (72.4)	29 (72.5)	33 (86.8)	13 (56.5)	13 (76.5)	0.129^F^
Time lag from onset of symptoms to FMDs diagnosis, years, mean (SD)		2.9 (3.5)^§^	4.6 (5.6)	6.4 (6.3)	5.1 (5.1)	4 (4.1)	**0.005**^K^
, median (IQR, Q1-Q3)		1.5 (0.75–5)	3 (1–6)	4.5 (2–9)	3 (2–7)	3 (1.5–4.5)	
FMD onset, *n* (%)							
Acute		51 (87.9)	24 (60)^**@**^	29 (76.3)	19 (82.6)	9 (52.9)^**@**^	**0.004**^F^
Slowly progressing		7 (12.1)	16 (40)	9 (23.7)	4 (17.4)	8 (47.1)	
FMD spontaneous remissions, (%)		33 (56.9)	21 (52.5)	19 (50)	16 (69.6)	7 (41.2)	0.429^C^
Diagnosis of FMDs, *n* (%)°							
General neurologist		23 (39.7)	3 (7.5)^**@**^	2 (5.3)^**@**^	2 (8.7)	4 (23.5)	<**0.001**^**F**^
Neurologist specialized in movement disorders		30 (51.7)	35 (87.5)^**@**^	34 (89.5)^**@**^	19 (82.6)	12 (70.6)	<**0.001**^**F**^
Self-reported non-motor symptoms, *n* (%)							
Anxiety		22 (37.9)	24 (60)	16 (42.1)	10 (43.5)	6 (35.3)	0.232^C^
Fatigue		21 (36.2)	9 (22.5)	13 (34.2)	6 (26.1)	4 (23.5)	0.565^C^
Pain		22 (37.9)	7 (17.5)	18 (47.4)^**#**^	5 (21.7)	5 (29.4)	**0.040**^C^
Migraine		11(19)	6 (15)	8 (21.1)	2 (8.7)	2 (11.8)	0.763^F^
Insomnia		12 (20.7)	10 (25)	5 (13.2)	2 (8.7)	5 (29.4)	0.325^F^
Panic attacks		8 (13.8)	5 (12.5)	5 (13.2)	4 (17.4)	0 (0)	0.535^F^
Depersonalization/derealization		7 (12.1)	2 (5)	4 (10.5)	4 (17.4)	0 (0)	0.322^F^
Non-neurological comorbidities, *n* (%)		26 (44.8)	20 (50)	7 (18.4))^**#**^	7 (30.4)	8 (47.1)	**0.029**^**C**^
Health status^§^, mean (SD)		1.8 (0.9)	1.8 (1.1)	1.4 (0.7)	1.4 (0.7)	1.7 (0.7)	0.735^K^
Median (IQR, Q1-Q3)		1.5 (1–2.25)	1 (1–2.75)	1 (1–2)	1 (1–2)	2 (1–2)	
Associated FND, *n* (%)							
Sensory functional symptoms		30 (51.7)^#§^*	3 (7.5)	2 (5.3)	5 (21.7)	2 (11.8)	**<0.001**^**F**^
Non-epileptic seizures		8 (13.8)	3 (7.5)	6 (15.8)	4 (17.4)	0 (0)	0.344^F^
Visual functional symptoms		4 (6.9)	2 (5)	0 (0)	0 (0)	3 (17.6)	0.063^F^
Cognitive functional symptoms		6 (10.3)	3 (7.5)	2 (5.3)	0 (0)	1 (5.9)	0.617^F^
Fibromyalgia		3 (5.2)	0 (0)	3 (7.9)	1 (4.3)	0 (0)	0.402^F^
Irritable bowel syndrome		3 (5.2)	1 (2.5)	0 (0)	1 (4.3)	1 (5.9)	0.565^F^
Psychiatric comorbidities, *n* (%)		18 (31)	12 (30)	13 (34.2)	9 (39.1)	4 (23.5)	0.864^C^
Predisposing factors, *n* (%)							
Childhood psychological trauma		3 (5.2)	1 (2.5)	2 (5.3)	1 (4.3)	1 (5.9)	0.943^F^
Childhood physical trauma		0 (0)	0 (0)	1 (2.6)	1 (4.3)	0 (0)	0.290^F^
Precipitating factors, *n* (%)							
Psychological trauma		14 (24.1)	8 (20)	15 (39.5)	4 (17.4)	1 (5.9)	0.073^F^
Physical trauma		5 (8.6)	2 (5)	8 (21.1)	2 (8.7)	1 (5.9)	0.217^F^
Surgery		7 (12.1)	2 (5)	6 (15.8)	3 (13)	3 (17.6)	0.500^F^

*FMDs* functional motor disorders, *FND* functional neurological disorders; ^°^Before enrollment, *SD* standard deviation, *IQR* interquartile range; statistical testing: one-way ANOVA (A), Kruskal–Wallis test (K), Chi-square test (C), Fisher’s exact test (F); significant values at *p* < 0.05 in bold. ^§^Health status = calculated as the average of total number of non-neurological comorbidities. Age = patient’s age at the enrollment in this study and our diagnosis of FMDs

Post-hoc comparisons, *p* < 0.05: ^#^vs tremor; *vs gait disorders; ^§^vs dystonia; ^@^vs weakness

^a^The education variable reported 19 missing

There were significant differences in terms of age between groups, with patients with tremor and gait disorders being older than the others (Table 1), whereas no differences were noted in terms of sex distribution.

Patients with functional weakness had more commonly an acute onset (87.9%) as compared with patients with tremor (60%, *p* < 0.05) and gait disorders (52.9%, *p* < 0.05), a shorter time lag from onset of symptoms and FMDs diagnosis (mean ± SD 2.9 ± 3.5, median 1.5, IQR 0.75–5; years) than patients with dystonia (mean ± SD 6.4 ± 6.3, median 4.5, IQR 2–9, years) and had more commonly associated sensory functional symptoms (51.7%) than patients with tremor (7.5%, *p* < 0.05), dystonia (5.3%, *p* < 0.05) and gait disorders (11.8%, *p* < 0.05) (Table 1).

Although patients with dystonia were less likely to have non-neurological comorbidities, the total number of comorbidities, which can be considered as a proxy of health status, did not differ between groups (Table 1). Patients with dystonia complained more often of associated pain (47.4%) than patients with tremor (17.5%, *p* < 0.05). The first diagnosis of FMDs, before the patients’ enrollment in this study, was performed by a movement disorder specialist in over 70% of patients in each phenotypic group except for functional weakness, which was diagnosed in 40% of the patients by a general neurologist. No other differences were noted between groups in terms of associated FNDs, self-reported non-motor symptoms, psychiatric comorbidities, predisposing and precipitating factors (Table 1).

## Discussion

In the current study, we aimed to compare several demographic and clinical variables in patients with different isolated FMDs phenotypes. Patients with FMDs often have multiple phenomenologies [5] and clinicians have to make a judgment on which one is “predominant”, likely introducing a bias when attempting to compare different phenotypes. To overcome this limit, we enrolled patients with isolated FMDs and without co-morbid neurological disorders, aiming to answer the question of whether they could represent different expressions of the same disorder or reflect distinct entities.

All groups displayed similar rates of psychiatric and other non-neurological comorbidities, associated FNDs, predisposing and precipitating factors and self-reported non-motor symptoms, with the exception of pain that, as expected [6, 7] was found to be more common in patients with functional dystonia than in patients with tremor, and of sensory functional symptoms that were commoner in patients with weakness than in patients with other phenotypes [8]. These results might support the concept that common mechanisms might underlie different FMDs, as also suggested by the frequent overlap of different FMDs in the same patient, as observed in a previous study [3, 7].

As mentioned above, patients with dystonia had higher rates of pain and this might be the result of a dissociation between the sensory-discriminative and cognitive-affective components of pain (increased pain tolerance together with a normal pain threshold) [7]. We also found that functional tremor and gait disorders were the most frequent FMDs in older patients which is in line with a study focusing on FMDs in the elderly population [9]. The reasons why this happens are unclear. As action tremor and gait disturbances (determined by neurological or orthopedic conditions) are common in this age group, we might speculate that exposure to these symptoms in the elderly population might be responsible of illness beliefs which are meant to be a crucial factor in FMDs generation [10]. A significant impact of disease modeling within the family or close friendship has been demonstrated in FMDs [11], and the same concept might be applicable in a wider sense considering the common knowledge that tremor, for instance due to Parkinson disease, and gait disturbances occur in elderly age.

In line with previous studies, patients with functional weakness were more likely to have an acute onset as well as associated sensory functional symptoms in the affected body region than patients with other phenotypes. Acute onset of functional weakness has been also reported by a previous study, in which different factors closely associated with the onset could be identified [12]. These included dissociative symptoms, with the hypothesis that functional weakness could therefore be a form of ‘hemi-depersonalisation’ or compartmentalisation, or physical injuries, in which the relationship with the functional weakness could be in terms of immobility and/or increased attention paid to a body part [12]. It is therefore important to evaluate associated factors at onset, as these might represent clues to identify the mechanism of the development of functional weakness [12].

The frequent co-occurrence of functional weakness and sensory symptoms is intriguing and might be explained by a common pathophysiological mechanism: hypoactive subcortical circuits controlling sensorimotor function might drive deficient voluntary motor behavior and sensory loss, similar to what happens in patients with motor neglect [8]. This is different from what is construed to occur in the context of functional symptoms characterized by excessive motor output, in which increased activation of areas implicated in self-awareness, self-monitoring, and active motor inhibition has been observed [13].

People with functional weakness also had a shorter diagnostic time lag and were diagnosed more frequently by general neurologists. Altogether, these features highlight that general neurologists are more familial with this type of clinical presentation and are more confident in performing the diagnosis of functional weakness in comparisons with other phenotypes. The consequence of this might be reflected by the longer diagnostic delay, especially for the group with functional dystonia, which call for an effort to increase knowledge about FMDs among general neurologists in view of the fact that diagnostic delay is a strong predictor of poor prognosis in this population [5].

We found comparable and low rates of predisposing factor as well as of psychiatric comorbidities between groups. It is conceivable that childhood psychological/physical trauma might have a weaker impact in patients with adult-onset FMDs, but we should also highlight that formal psychological and psychiatric evaluations were not performed in our study. This might have led to an underestimation of psychological trauma and psychiatric disturbances in comparison to previous studies [3, 14]. Supporting this hypothesis, we note that, for instance, self-reported anxiety was more common than a formal diagnosis of mood disorder made by a psychiatrist in all FMDs groups. However, even in case of higher rates of a formal psychiatric diagnosis, one would expect them to evenly distribute across different phenotypes, thus not explaining why one patient might develop one or another motor disturbance. Precipitating factors were statistically comparable among phenotypes, even though patients with dystonia showed higher rates of both physical and psychological trauma. Whereas the link between physical trauma and dystonia in the same body part seems more straightforward, the relationship between psychological trauma and dystonia is less clear. A previous study also found that patients with functional dystonia had high rates of psychological stressors [15], which reinforces the need for a comprehensive evaluation of these patients.

We acknowledge some limitations. Although we identified a large overlap between different FMDs phenotypes, our comparisons are only based on clinical features, which prevents drawing definitive conclusions about common pathophysiological mechanisms. Therefore, future studies, using for instance neurophysiological and imaging techniques, are needed to deepen the pathophysiological mechanisms of FMDs [16]. Furthermore, our data cannot answer the question about why some patients may develop a phenotype instead of another. A prior study reported some gender-driven differences between FMDs phenotype, with functional dystonia being more common in females and a trend for gait disturbance being commoner in males [17]. However, it is likely that additional features drive the development of a specific phenotype.

We also note that recruitment was performed in tertiary-care, movement disorder centers and a proportion of patients, for instance with functional weakness, might have not been referred to the participating centers. Moreover, for the aim of the current study we only selected patients with isolated FMDs. For these reasons, prevalence figures of different phenotypes reported in this study should not be intended as representative of the entire population of FMDs.

Moreover, we could not determine the severity of recorded symptoms as we did not employ any rating instrument for them (i.e. pain). Finally, the frequency of psychological stressors might be underestimated because of the failure of the providers not to ask to specific questions. In-clinic interviews are prone to ascertainment bias as demonstrated by the detection of higher rates of psychological stressors in anonymous surveys [18]. Previous studies have reported a higher frequency of psychological stressors including physical abuse and sexual assault in FMDs compared to our study [19], but we are unaware of any research looking at possible differences between phenotypes. Future prospective studies are therefore needed to clarify how these factors differ among different FMDs phenotypes to further develop specific management strategies.

In summary, our data confirm the evidence of a large overlap between FMDs phenotypes. If the clinical categorization according to the main motor phenotype remains valuable to guide the diagnostic work-up, it might not be useful to speculate about the underlying pathophysiology nor to categorize and measure outcome in FMDs phenotypes. This finding is supported by the significant overlap in terms of multiple non-motor symptoms which might be equally detrimental to patients’ functioning and quality of life and should be addressed in all FMDs patients [3]. The difference in the age at onset between groups requires future studies to explain the mechanisms driving the development of a certain phenotype, upon which planning of treatment options is partly dependent [20, 21].

## Supplementary Information

Below is the link to the electronic supplementary material.

Supplementary file1 (DOCX 16 KB)
